# Why Is There Low Utilization of Biosimilars in Inflammatory Bowel Disease Patients by Gastroenterology Advanced Practice Providers?

**DOI:** 10.1093/crocol/otab004

**Published:** 2021-03-27

**Authors:** Nana Bernasko, Kofi Clarke

**Affiliations:** Department of Medicine, Penn State Hershey Medical Center, Division of Gastroenterology and Hepatology, Hershey, Pennsylvania, USA

**Keywords:** safety, providers, treatment, Crohn disease, ulcerative colitis, biosimilars

## Abstract

**Aim:**

To assess knowledge, practice patterns and attitudes toward the use of biosimilars by Advanced Practice Providers (APPs) treating patients with inflammatory bowel disease (IBD).

**Background:**

APPs provide care in a variety of healthcare settings including medical specialties. In Gastroenterology, they are an integral part of providing care to a complex group of patients with IBD. There has been an increase in options of medical therapies for treating IBD. These include small molecules, biologics, and biosimilars. Adoption of biosimilars for treatment of IBD patients by gastroenterologists in the United States compared to Europe has been slow for several reasons. There is lack of data on their use by APPs who provide frontline IBD clinical care in the United States.

**Methods:**

Questionnaire-based survey of APPs attending Gastroenterology conferences with a focus on IBD.

**Results:**

APPs in gastroenterology do not routinely consider the use of biosimilars in their practice.

**Conclusions:**

There is low utilization of biosimilars in treating IBD patients by APPs. In addition, there are significant concerns about risk of side effects as well as perceived lack of APP targeted educational resources.

## INTRODUCTION

Nurse practitioners and physician assistants, collectively known as Advanced Practice Providers (APPs), emerged as part of the response to the shortage of physicians in the United States in the mid-nineties.

APPs provide care in a variety of healthcare settings including medical specialties. The U.S. Bureau of Labor Statistics (BLS) data in 2016 indicate there were 106,200 physician assistant jobs and 155,500 nurse practitioner jobs.^1,2^ APPs examine, diagnose, and treat patients either as independent practitioners, in some states (NPs), or under direct physician supervision. They are an integral and essential part of providing primary and specialty care. Data from The Agency for Healthcare Research and Quality (AHRQ) in 2010 estimated that 48% of nurse practitioners and 57% of physician assistants practice in subspecialty care.^3^

There are several gastroenterology practices which incorporate APPs in their care teams to help improve access to specialty care. In 2008, approximately 25.1% of gastroenterology practices employed APPs and by 2016, this had increased to 28.7%.^4^ This number is expected to grow with the projected shortage of 1050 physicians in gastroenterology by 2020.^5^

As a result of state regulations, and the needs of individual subspecialty practices, the roles of APPs vary greatly within gastroenterology practices. The extensive array of clinical services they provide include consultation, diagnoses and treatment, ordering, performing and interpreting tests, prescribing medications, and inpatient rounding.

Treatment options in inflammatory bowel disease (IBD) have expanded significantly in the past several years. More recently, the use of medical therapies including biologics and small molecules have increased. With this increase has come the realization of the cost implications of these newer therapies.

After successful introduction in Rheumatology, biosimilars have been introduced for the care of patients with IBD. Biosimilars are biological products that have no clinically meaningful differences from, and are highly similar to, existing FDA-approved products.^6^ Biosimilars were developed in an effort to reduce the costs of originator biologic drugs and should not be confused with a generic product.^7^ In clinical practice, they may be relatively less expensive and have been in use in Rheumatology both in the United States and in Europe. Most of the data on the use of IBD biosimilars, from a safety and efficacy standpoint, have come from Europe as they have been available and in use there for several years. The general consensus from these studies is that biosimilars seem to have similar efficacy, safety, and immunogenicity as the originator product.^8^ In addition, the recent review by Scott et al surmised that the available data up to 2017 indicate similar efficacy, safety, and immunogenicity comparing the approved infliximab and adalimumab biosimilars to the originator molecules.^9^

Furthermore, although inadequately powered to detect noninferiority within individual diseases, the NOR-SWITCH extension study did not show a difference in safety and efficacy between patients maintained on CT-P13 (an infliximab biosimilar) and patients who switched from originator infliximab to CT-P13.^10^

The 10 biosimilars that have been approved in the United States as options of care for IBD patients are listed in Table 1.^11^ Of these approved biosimilars, only 2 infliximab biosimilars have been marketed in the United States. The adalimumab biosimilars seem to be still in patent litigation.^8^ To our knowledge, however, there are no existing data on their use, prescription patterns, and level of familiarity of APPs who provide significant frontline care in gastroenterology practices. We conducted a survey to assess gastroenterology APPs perspectives, practice patterns, and use of biosimilars.

**TABLE 1.** List of Available Biosimilars and Their FDA Approval Status in the United States

Originator Product	Biosimilar	FDA Approval	Marketing Status
Humira	Hulio (adalimumab-fkjp)	July 6, 2020	Not available
	Abrilada (adalimumab-afzb)	November 15, 2019	Not available
	Hadlima (adalimumab-bwwd)	July 23, 2019	Not available
	Hyrimoz (adalimumab-adaz)	October 30, 2018	Not available
	Cyltezo (adalimumab-adbm)	August 25, 2017	Not available
	Amjevita (adalimumab-atto)	September 23, 2016	Not available
Remicade	Avsola (infliximab-axxq)	December 6, 2019	Not available
	Ixifi (infliximab-qbtx)	December 13, 2017	Not available
	Renflexis (infliximab-abda)	April 21, 2017	July 2017
	Inflectra (infliximab-dyyb)	April 5, 2016	April 2016

## METHODS

A 9-question survey was created and administered via Survey Monkey to APPs attending 2 APP-dedicated, CME accredited Gastroenterology conferences over a 4-month period. Data on number of IBD patients seen per week, practice setting, level of knowledge of biosimilars as well as perceived access to resources to learn about biosimilars were collected. In addition, we sought to evaluate the likelihood of respondents prescribing a biosimilar in the ensuing year. To avoid duplication, APPs who had completed the survey at the initial conference in Chicago (Principles of Gastroenterology for the NP and PA), were excluded from participation at the second conference (Inflammatory Bowel Disease Update for Advanced Practice Clinicians and Allied Health Professionals), in Pennsylvania. There were no identifying data collected. Responses are summarized as percentages.

## RESULTS

Seventy-six APPs out of 277 (27.4%) conference attendees responded to the survey; greater than half of the respondents were working in a private practice setting.

Over 70% of respondents had never prescribed a biosimilar despite significant involvement in the care of or exposure to IBD patents.

**FIGURE 1.**
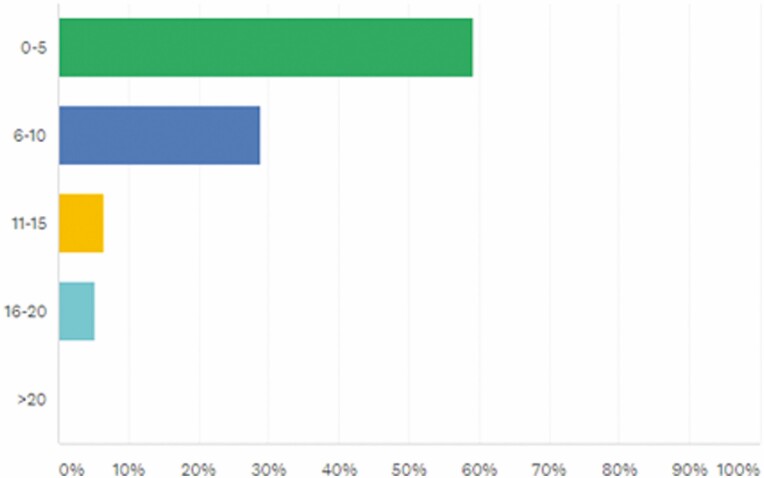
How many IBD patients do you see in a week? Answered: 76, Skipped: 0.

Figure 1 summarizes the number of patients seen in a week by APPs.

**FIGURE 2.**
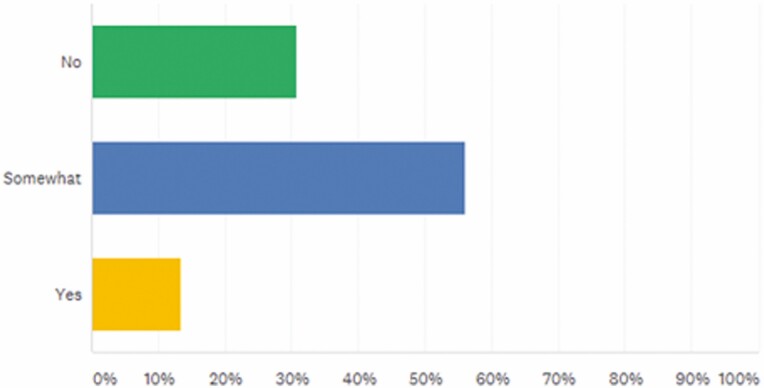
Do you feel you have enough resources to learn about biosimilars? Answered: 75, Skipped: 1.

Less than 6% of respondents were very knowledgeable on the use of biosimilars; 53.95% were somewhat knowledgeable and 40.79% were not knowledgeable (Fig. 2).

**FIGURE 3.**
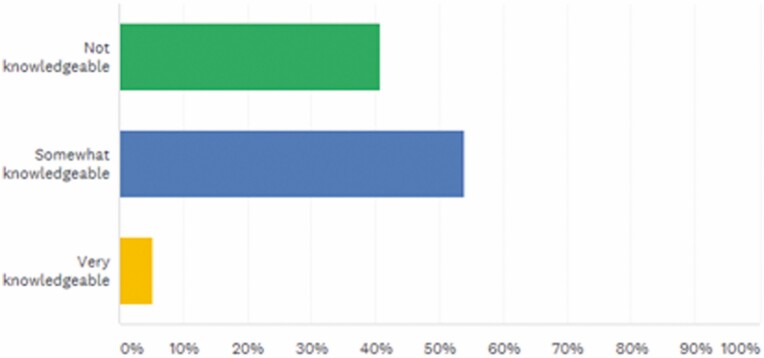
How would you rate your knowledge on biosimilars in the treatment of Inflammatory Bowel Disease (IBD)? Answered: 76, Skipped: 0.

Less than 1 in 6 respondents (13.33%) considered availability of resources targeted to APPs to learn about biosimilars as adequate (Fig. 3).

**FIGURE 4.**
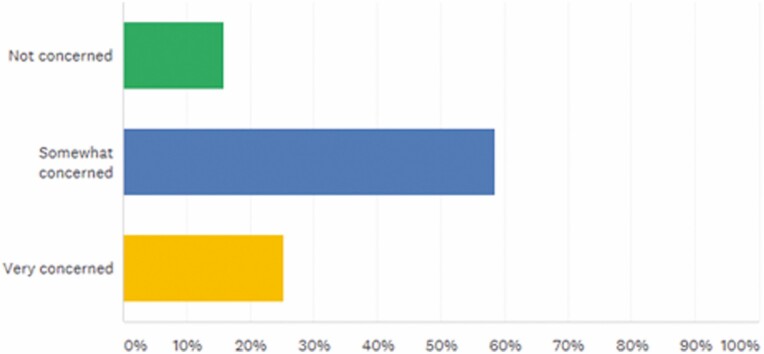
How concerned are you about the effectiveness of biosimilars in IBD? Answered: 75, Skipped: 1.

Regarding safety, 78.16% of respondents were somewhat concerned, or very concerned about biosimilar use in IBD. Furthermore, only 16% of respondents were not concerned about the effectiveness of biosimilars (Fig. 4).

**FIGURE 5.**
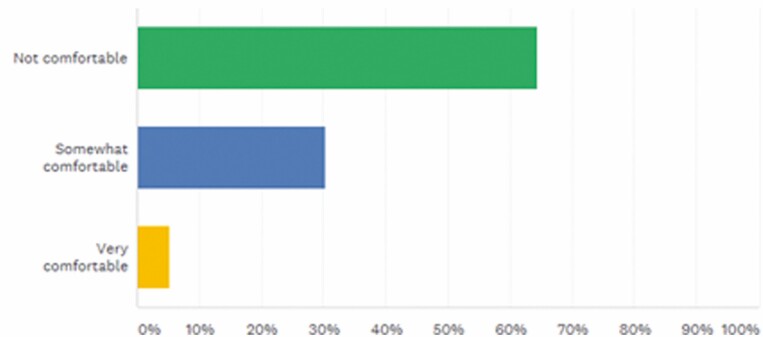
How comfortable are you switching patients from originator molecules to biosimilars in IBD? Answered: 76, Skipped: 0.

In reporting their comfort levels in switching patients from the originator molecules to biosimilars, only 5.26% were very comfortable (Fig. 5).

**FIGURE 6.**
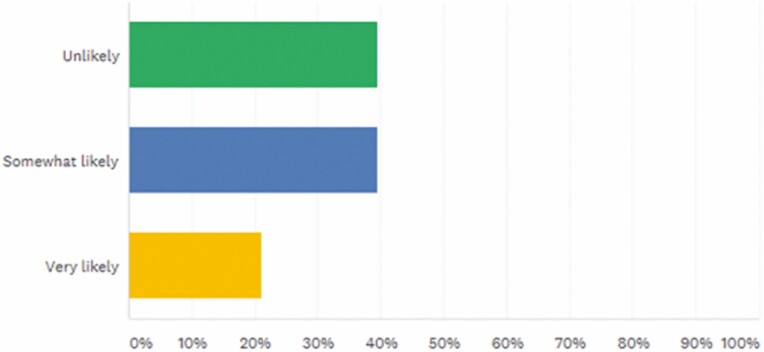
How likely are you to prescribe biosimilars in the next year? Answered: 76, Skipped: 0.

When asked how likely they were to prescribe biosimilars in the next year, 39.47% reported unlikely, 39.47% reported somewhat likely, and 21.05% reported very likely (Fig. 6).

**FIGURE 7.**
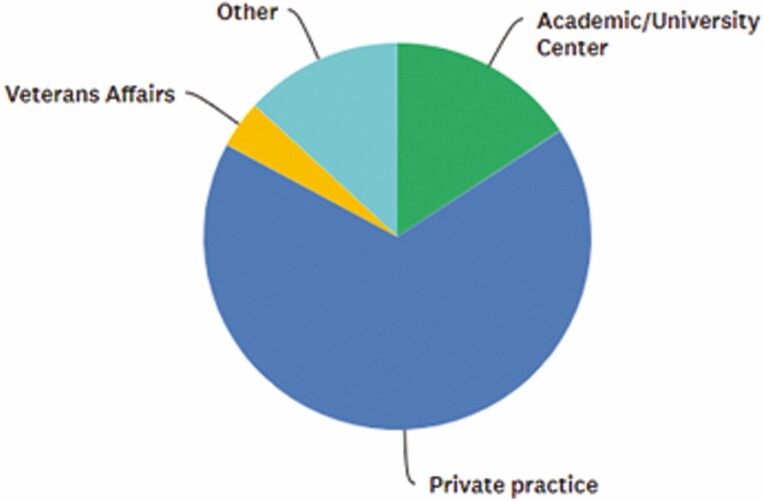
Which of the following best describes your practice setting? Answered: 76, Skipped: 0.

Figure 7 summarizes the different practice settings of the respondents.

## DISCUSSION

APPs use biosimilars very infrequently, and have significant concerns about prescribing biosimilars. Most importantly, their responses indicate a lack of perceived access to sources of information on biosimilars as well as having several reservations about safety and efficacy. APPs who attended the 2 conferences with an IBD focus may be more representative of those interested in IBD. As such there may be a selection bias of respondents. We postulate that the problem may be larger than assessed considering the broader APP pool who may not have a specific focus on or interest in IBD. The perceived lack of available resources targeted at APPs to learn about biosimilars is surprising and needs addressing. Perhaps future IBD and Gastroenterology conferences should include more breakout sessions with a focus on APPs as well as provide direction on resources for information. Our results shed some light on the extent of the problem and provide some baseline data on needs assessment to help guide topic selection at future conferences.

There are some limitations to our study. It is a questionnaire-based survey with respondents from 2 specific settings, and the results may not be generalizable. The response rate of 27.4% is within range of other survey-based studies. Although our total number of respondents was relatively small, it highlights a trend which requires further evaluation and investigation.

Despite the limitations, we believe our responsive audience was broad based from participants at 2 large conferences with a focus on IBD who provide care in a variety of practice settings.

Further studies are needed to better understand this observation. With additional data from other studies, specific targeted programs could be developed in conjunction with the professional societies to address the identified concerns. Furthermore, it is important to develop targeted training to improve the knowledge base of a large section of the healthcare provider work force who provide frontline care of IBD patients.

## Data Availability

Data not publicly available. No new data were created or analyzed.

## Notes

Funding: There was no funding for this project.

## References

[1] Bureau of Labor Statistics, U.S. Department of Labor. Occupational Outlook Handbook, Nurse Anesthetists, Nurse Midwives, and Nurse Practitioners. https://www.bls.gov/ooh/healthcare/nurse-anesthetists-nurse-midwives-and-nurse-practitioners.htm (18 January 2020, date last accessed).

[2] Bureau of Labor Statistics, U.S. Department of Labor. Occupational Outlook Handbook, Physician Assistants. https://www.bls.gov/ooh/healthcare/physician-assistants.htm (18 January 2020, date last accessed).

[3] Agency for Healthcare Research and Quality. Primary Care Workforce Facts and Stats No. 2. The Number of Nurse Practitioners and Physician Assistants Practicing Primary Care in the United States. https://www.ahrq.gov/sites/default/files/publications/files/pcwork2.pdf (18 January 2020, date last accessed).

[4] Martsolf GR , Barnes H, Richards MR, et al. Employment of advanced practice clinicians in physician practices. JAMA Intern Med. 2018;178:988–990. 29710094 10.1001/jamainternmed.2018.1515 PMC6126674

[5] Moses RE , McKibbin RD. Non-physician clinicians in GI practice part 1: current status and utilization. Am J Gastroenterol. 2017;112:409–410. 28220782 10.1038/ajg.2017.16

[6] U.S. Food & Drug Administration. Biosimilar and Interchangeable Products. https://www.fda.gov/Drugs/DevelopmentApprovalProcess/HowDrugsareDevelopedandApproved/ApprovalApplications/TherapeuticBiologicapplications/Biosimilars/ucm580419.htm (18 January 2020, date last accessed).

[7] Lichtenstein GR . Highlights in biosimilars from the world congress of gastroenterology at ACG 2017: introduction. Gastroenterol Hepatol. 2017;12:4–5.

[8] Rudrapatna VA , Velayos F. Biosimilars for the treatment of inflammatory bowel disease. Pract Gastroenterol. 2019;43:84–91. 31435122 PMC6703165

[9] Scott IF , Lichtenstein GR. Biosimilars in the treatment of inflammatory bowel disease: supporting evidence in 2017. Curr Treat Options Gastroenterol. 2018;16:147–164. 29492747 10.1007/s11938-018-0177-z

[10] Goll GL , Jorgensen KK, Sexton J, et al. Long-term efficacy and safety of biosimilar infliximab (CT-P13) after switching from originator infliximab: open-label extension of the NOR-SWITCH trial. J Intern Med. 2019;285:653–669. 30762274 10.1111/joim.12880 PMC6850326

[11] Drugs.com. How Many Biosimilars Have Been Approved in the United States? https://www.drugs.com/medical-answers/many-biosimilars-approved-united-states-3463281/ (13 August 2020, date last accessed).

